# Effects of Probiotic Supplementation on Circadian Rhythm‐Related Gene Expression and Body Weight in Mice With Disturbed Circadian Rhythms

**DOI:** 10.1002/mnfr.70579

**Published:** 2026-08-06

**Authors:** Büşra Tepebaşı, Mehtap Ünlü Söğüt, Menşure Nur Çelik, Sevtap Kabalı

**Affiliations:** ^1^ Department of Nutrition and Dietetics Graduate Education Institute, Ondokuz Mayıs University Samsun Türkiye; ^2^ Department of Nutrition and Dietetics Faculty of Health Sciences, Ondokuz Mayıs University Samsun Türkiye

**Keywords:** *Bmal1*, circadian rhythm, *Clock*, *Per2*, probiotic

## Abstract

Given the limited knowledge of probiotic effects on clock genes, this study investigated whether probiotics that regulate gut integrity and microbiota balance influence central circadian clock gene expression. In this study conducted on three groups [control group (CG), shifted group (SG), and shifted and probiotic supplement group (SPG)] of eight BALB/c mice each, Clock, Bmal1, and Per2 gene expressions and weight gain were evaluated. Although body weights at baseline, week 8, and week 16 were similar among groups, weight gain over time was significantly higher in the SG and SPG groups (*p*<0.05). Significant differences were observed between CG and SG at week 8 and between SG and SPG at week 16. Gene expression analyses revealed no significant differences in Clock expression. However, Bmal1 expression at ZT18 was significantly different between the CG and SPG groups (*p* = 0.045). These findings demonstrate the regulatory effect of probiotic supplementation on circadian disruption and weight gain, suggesting a potential role in circadian rhythm management. Nevertheless, the results should be interpreted cautiously because they are based on limited gene expression changes and require confirmation in larger studies.

## Graphical abstract

Male BALB/c mice were assigned to control, circadian disruption, or circadian disruption plus probiotic groups for 16 weeks. Body weight and circadian clock gene expression (Clock, Bmal1, and Per2) were evaluated. Probiotic supplementation partially modulated weight gain and influenced circadian gene regulation, supporting its potential as an adjunct strategy for circadian rhythm disorders.

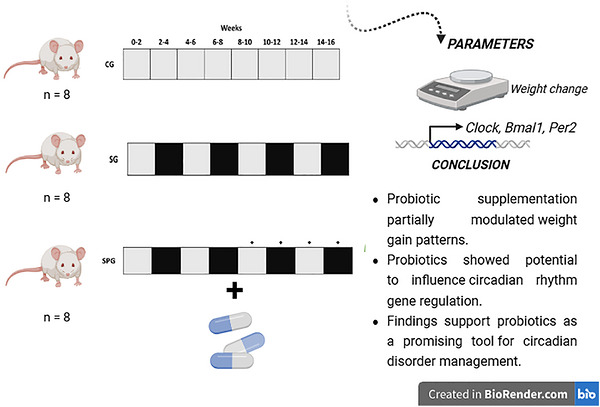

**Abbreviations**
CG: Control group
Cry: Cryptochrome
SCN: suprachiasmatic nucleus
SG: Shifted group
SPG: Shifted and probiotic supplement group
ZT: Zeitgeber Time

## 1 Introduction

Circadian rhythms are endogenous biobehavioral cycles that last approximately 24 h and are generated and regulated by the hypothalamic suprachiasmatic nucleus (SCN), the circadian master/central clock [1]. Circadian rhythms are generated by a set of circadian clock genes found in nearly every cell of mammals, which direct the circadian expression of numerous clock‐controlled genes [2, 3].

At the cellular level, the circadian clock rhythm is governed by a series of genes and proteins that form interlocking positive and negative feedback loops. These genes consist of the transcription factors “Clock” and “Bmal1” and their targets, the genes Period (Per 1,2,3) and Cryptochrome (Cry1 and Cry2) [4]. These clock gene heterodimers essentially establish an autoregulatory transcriptional‐translational feedback loop by acting as transcriptional activators and repressors of one another [5].

Circadian disruption is defined as a disruption in biological timing that can occur at different organizational levels, from molecular rhythms in cells to misalignment of behavioral cycles with environmental changes [6]. Disruption of the circadian rhythm is associated with many diseases affecting almost all systems of the body, including the neurological, immune, and gastrointestinal systems [7]. Disrupted circadian rhythm leads to higher or lower expression levels of many clock genes and associated target genes [8, 9].

There is a bidirectional interaction between the circadian rhythm and the intestinal microbiota; it has been observed that circadian rhythm disruption leads to intestinal microbiota dysbiosis, and at the same time, the intestinal microbiota affects the circadian rhythm [10]. Dysbiosis resulting from circadian rhythm disruption acts as a trigger for increased intestinal permeability and inflammation, and circadian‐induced changes in microbiota composition are thought to play a role in mediating the detrimental effects of circadian dysregulation [11, 12]. It is known that probiotics can regulate intestinal epithelial integrity, prevent intestinal barrier destruction, regulate the mucosal immune system and correct dysbiosis by modulating the intestinal microbiota [13, 14] Although a recent study demonstrated the effect of prebiotic supplementation on circadian rhythm genes [15], a literature review revealed that studies investigating the effects of probiotic supplementation on circadian rhythm gene expression in experimental animals are limited. This study aimed to investigate the effects of probiotic supplementation on central circadian rhythm clock genes in mice with disrupted circadian rhythms.

## 2 Experimental Section

### 2.1 Experimental Animals and Study Design

In this study, 24 male BALB/C mice aged 8–10 weeks were used. All procedures performed on the animals were conducted in accordance with ARRIVE guidelines and approved by the Ondokuz Mayıs University Local Ethics Committee for Laboratory Animals (Number: E‐68489742‐604.01.03‐97230; Date: 27.07.2021). Animal welfare was monitored throughout the study, and the 3R principle (replacement, reduction, and refinement) was strictly applied. The animals were housed in separate polycarbonate cages with stainless steel lids, with ad libitum access to a standard pellet diet and normal tap water. Prior to the experiments, the animals were acclimated to the cage conditions, ambient temperature, and humidity for 1 week. Three experimental groups were formed, each consisting of eight mice, and the mice were randomly assigned to the groups using a simple randomization method. Outcome assessments and data analyses were performed by investigators blinded to the group allocation. No animals were excluded from the study, and all animals completed the experimental protocol. The experimental groups are as follows:

▪ Control group (CG): No intervention was performed (*n* = 8).
▪ Shifted group (SG): Mice with disrupted circadian rhythms (*n* = 8).
▪ Shifted and probiotic supplement group (SPG): Mice with disrupted circadian rhythms that received probiotic supplementation (*n* = 8).

### 2.2 Circadian Manipulation

During the study, the humidity (55 ± 10%) and temperature (22 ± 2°C) values of the environment in which the animals were kept were maintained constant throughout the entire 16‐week experimental period. Environmental conditions, including room temperature, humidity, ventilation, and light intensity, were monitored regularly to ensure stability during all circadian manipulation procedures. For mice in CG, light (300–400 lux) was turned on at 08:00 and off at 20:00, providing a normal 12‐h light/dark cycle for 16 weeks. In the control condition, Zeitgeber Time (ZT) 0 was defined as the time of lights on (08:00), ZT6 as 14:00, ZT12 as the time of lights off (20:00), and ZT18 as 02:00.

For the SG and SPG groups, the animals were initially maintained under a standard 12‐h light/12‐h dark cycle for the first two weeks (lights on at 08:00 and lights off at 20:00). Beginning on day 15, the light/dark schedule was reversed by maintaining the room in complete darkness during the usual light phase (08:00–20:00) and subsequently switching the light phase to the opposite period. This reversed light/dark cycle was maintained for 2 weeks before being switched again to the original schedule. Accordingly, alternating normal and reversed light/dark cycles were repeated every 2 weeks throughout the 16‐week experimental period. During dark phases, a specially designed light‐isolated room with blackout windows was used to prevent unintended light exposure and external light contamination. For the circadian rhythm‐disrupted groups exposed to the reversed light/dark cycle, ZT values were defined according to the inverted lighting schedule, where ZT0 represented lights on at 20.00, ZT6 at 02.00, ZT12 represented lights off at 08:00, and ZT18 at 14:00. Thus, ZT points in the shifted groups were determined relative to the reversed light/dark cycle.

**FIGURE 1:**
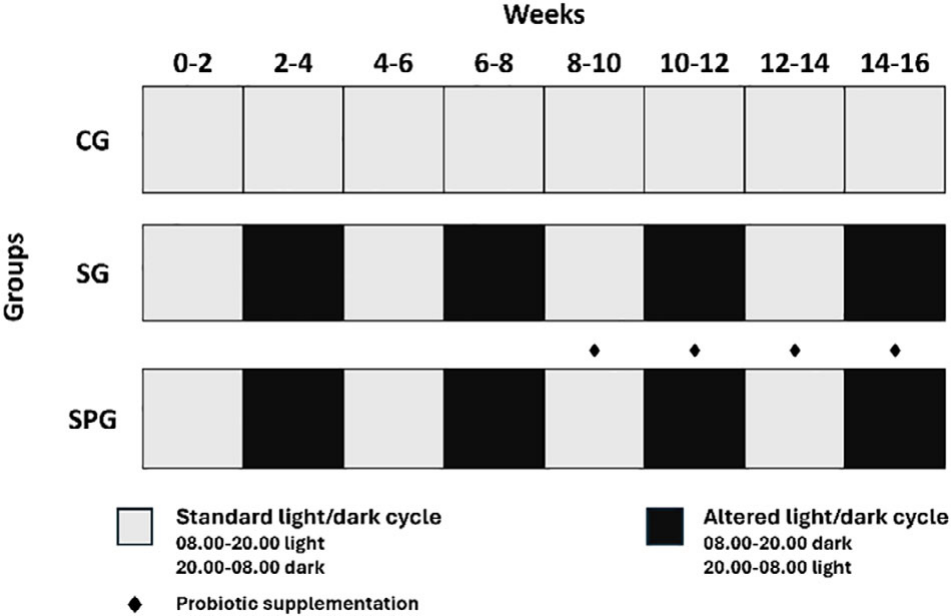
Protocol for altering the light/dark cycle. CG: Control group, SG: Shifted group, SPG: Shifted and probiotic supplement group (*n* = 8 mice per group).

Additionally, the mice in the SPG group received probiotic supplementation orally via gavage every day of the week, including weekends, starting from the 8th week and continuing throughout the experimental period. To minimize procedural variability, probiotic administration was performed at approximately the same time each day by the same investigator using a standardized gavage protocol. The experimental protocol is shown in Figure 1.

### 2.3 Probiotic Supplementation

VSL#3 was used as the probiotic in this study. VSL#3 contains *Streptococcus thermophilus* BT01, *Lactobacillus plantarum* BP06, *Lactobacillus acidophilus* BA05, *Lactobacillus helveticus* BD08, *Lactobacillus paracasei* BP07, *Bifidobacterium animalissubsp*. lactis BL03, *Bifidobacterium animalissubsp*. lactis BL04, and *Bifidobacterium breve* BB02, formulated at 63 mg per strain. The probiotic was stored at 2°C–8°C. Since the regulatory effects of VSL#3 on adverse outcomes caused by circadian rhythm disorders have been demonstrated in animal models, VSL#3 was chosen for this study, and the probiotic supplement was administered orally via gavage at a dose of 1 × 10^9^ colony‐forming unit (CFU) daily to mice in the SPG starting from the 8th week [16, 17]. The administered dose was based on the CFU content provided by the manufacturer. Independent microbiological confirmation of CFU viability at the time of administration was not performed, which should be considered a methodological limitation.

Probiotic administration was performed continuously on a daily basis, including weekends, to ensure consistent exposure throughout the circadian rhythm intervention period. The same gavage volume and administration procedure were applied throughout the study to maintain dosing consistency among animals. Because body weight differences between groups were relatively limited during the intervention period, probiotic dosing was administered as a fixed daily CFU amount rather than normalized individually according to body weight.

### 2.4 Assessment of Body Weight

Body weights of the animals were measured at the beginning, at 8 weeks, and at 16 weeks using a 0.1 g sensitive balance. The body weight progression was additionally evaluated by separating the experimental period into preintervention (baseline to week 8) and postintervention (week 8 to week 16) phases in order to assess whether probiotic supplementation modified the trajectory of weight gain after initiation of the probiotic treatment.

### 2.5 RNA Isolation and Quantitative RT‐PCR

After completion of the experimental period, the animals were divided into groups of two mice from each group throughout the daily cycle, at specified intervals every 6 h [ZT0 (lights on), ZT6, ZT12, and ZT18], with two mice from each group sampled at each time point. For the SG and SPG groups, ZT points were determined based on a reversed light/dark schedule; in both normal and reversed cycles, ZT0 represented when the lights were on, and ZT12 represented when the lights were off. Thus, biological replicates consisted of two independent animals per group at each ZT point, and different animals were sacrificed at each ZT point. Therefore, repeated sampling from the same animal was not performed during circadian gene expression analyses. Following sacrifice, brain tissues were removed and dissected rapidly. All tissues were frozen and stored at −80°C. Total RNA was extracted from brain tissue using TRIzol reagent (Invitrogen, USA) according to the manufacturer's instructions. RNA samples were measured using a spectrophotometer and reverse transcribed into cDNA using a commercial kit according to the manufacturer's protocol. Real‐time quantitative PCR was performed using previously published primers in RT‐PCR systems according to the kit protocol. The primer sequences are shown in Table 1. The Delta‐Delta (2–∆∆Ct) method was used to analyze the expression of target genes normalized to the reference gene GAPDH.

**TABLE 1:** Primer sequences for real‐time PCR.

Genes	Primer sequence	References
*Clock*	F: 5′‐CACAGGCCAGCACATGAT‐3′	[33]
	R: 5′‐CACTCATTACACTCTGTTGACTCTGA‐3′	
*Per2*	F: 5′‐GTTCCAGGCTGTGGATGAA‐3′	[33]
	R: 5′‐GGCGTCTCGATCAGATCCT‐3′	
*Bmal1*	F: 5′‐ATTCCAGGGGGAACCAGA‐3′	[33]
	R: 5′‐GAAGGTGATGACCCTCTTATCCT‐3′	
GAPDH	F: 5′‐CATCACTGCCACCCAGAAGACTG‐3′	[34]
	R: 5′‐ATGCCAGTGAGCTTCCCGTTCAG‐3′	

At each ZT point (ZT0, ZT6, ZT12, and ZT18), brain tissues from two biological replicates (*n* = 2 mice per group) were analyzed for gene expression experiments. Accordingly, the total number of animals used for circadian gene expression analyses corresponded to the eight animals originally assigned to each experimental group, distributed across the four ZT points (*n* = 2 independent mice per ZT point). QRT‐PCR measurements were performed in technical triplicate for each biological sample, and the average Ct values were used for statistical analyses. Therefore, statistical analyses for gene expression data were based on biological replicates (*n* = 2 mice per group per time point), whereas technical replicates were used to improve analytical reliability. Because each animal contributed data to only one ZT point, observations were treated as independent samples in the statistical analyses. Because of the limited number of biological replicates at each time point, the gene expression findings should be considered exploratory and interpreted with caution.

### 2.6 Statistical Analyses

The sample size and group design of the study were determined using the G‐Power version 3.1 (Kiel, Germany) program (*α* = 0.05 and *β* = 95%) [18]. Statistical analyses were performed using GraphPad Prism version 10.0 (California, USA). Data are presented as mean ± standard deviation (SD) for descriptive analyses and graphical presentations. In addition, individual animal values were displayed in graphical presentations to improve visualization of data distribution and biological variability within experimental groups.

Body weight measurements obtained longitudinally from the same animals at baseline, week 8, and week 16 were analyzed using a two‐way repeated‐measures ANOVA, with time as the within‐subject factor and experimental group as the between‐subject factor. Bonferroni‐adjusted post hoc multiple‐comparison tests were applied when appropriate to control for type I error across repeated pairwise comparisons. To further evaluate the effect of probiotic supplementation on the progression of body weight changes, the experimental period was additionally separated into preintervention (baseline to week 8) and postintervention (week 8 to week 16) phases. Body weight gain trajectories during these intervals were compared between groups to determine whether probiotic supplementation modified weight gain progression after circadian rhythm disruption.

For circadian rhythm gene expression analyses, independent biological samples were collected at each ZT point (ZT0, ZT6, ZT12, and ZT18), with two animals per group analyzed at each time point (*n* = 2 biological replicates per group per ZT point). Because different animals were sacrificed at each ZT point, qRT‐PCR data did not constitute a repeated‐measures design.

Gene expression data were analyzed using two‐way ANOVA with experimental group and ZT as fixed factors, followed by Bonferroni‐adjusted post hoc testing for multiple comparisons across groups and time points. However, due to the limited number of biological replicates per time point, these analyses were considered exploratory and interpreted cautiously. The limited sample size restricted robust assessment of normality and homogeneity of variance assumptions and reduced the statistical power to detect subtle circadian expression differences. Technical triplicates were averaged prior to statistical analysis, and only biological replicates were considered as independent observations in the statistical models. Because each ZT point consisted of independent biological samples obtained from different animals, no repeated‐measures or mixed‐effects model was applied for qRT‐PCR analyses. A *p*‐value < 0.05 was considered statistically significant.

## 3 Results

The baseline, eighth week, and 16th week (final) weights of mice in the CG, SG, and SPG groups are shown in Table 2. The average baseline weights of the mice in the CG, SG, and SPG groups were 37.5 ± 2.77, 38.12 ± 1.55, and 37.0 ± 3.58 g, respectively. The average weights of the mice in the groups at eighth week were 38.1 ± 1.7 g in the CG group, 39.5 ± 0.7 g in the SG group, and 38.7 ± 4.6 g in the SPG group. The final average weights were 38.8 ± 1.5 g in the CG group, 41.3 ± 1.1 g in the SG group, and 39.0 ± 3.8 g in the SPG group.

**TABLE 2:** Evaluation of body weights of experimental groups.

	CG (*n* = 8) x̄ ± SD	SG (*n* = 8) x̄ ± SD	SPG (*n* = 8) x̄ ± SD	*p*
Body weight (g)				
Baseline	37.5 ± 2.7	38.1 ± 1.5	37.0 ± 3.5	0.311
8th week	38.1 ± 1.7	39.5 ± 0.7	38.7 ± 4.6	0.059
16th week	38.8 ± 1.5^a^	41.3 ± 1.1^b^	39.0 ± 3.8^a^	**0.013**

^a, b^Shows statistical difference between groups (*p* < 0.05). Different letters indicate significant difference, while the same letters indicate no difference. CG: Control group, SG: Shifted group, SPG: Shifted and probiotic supplement group.

**FIGURE 2:**
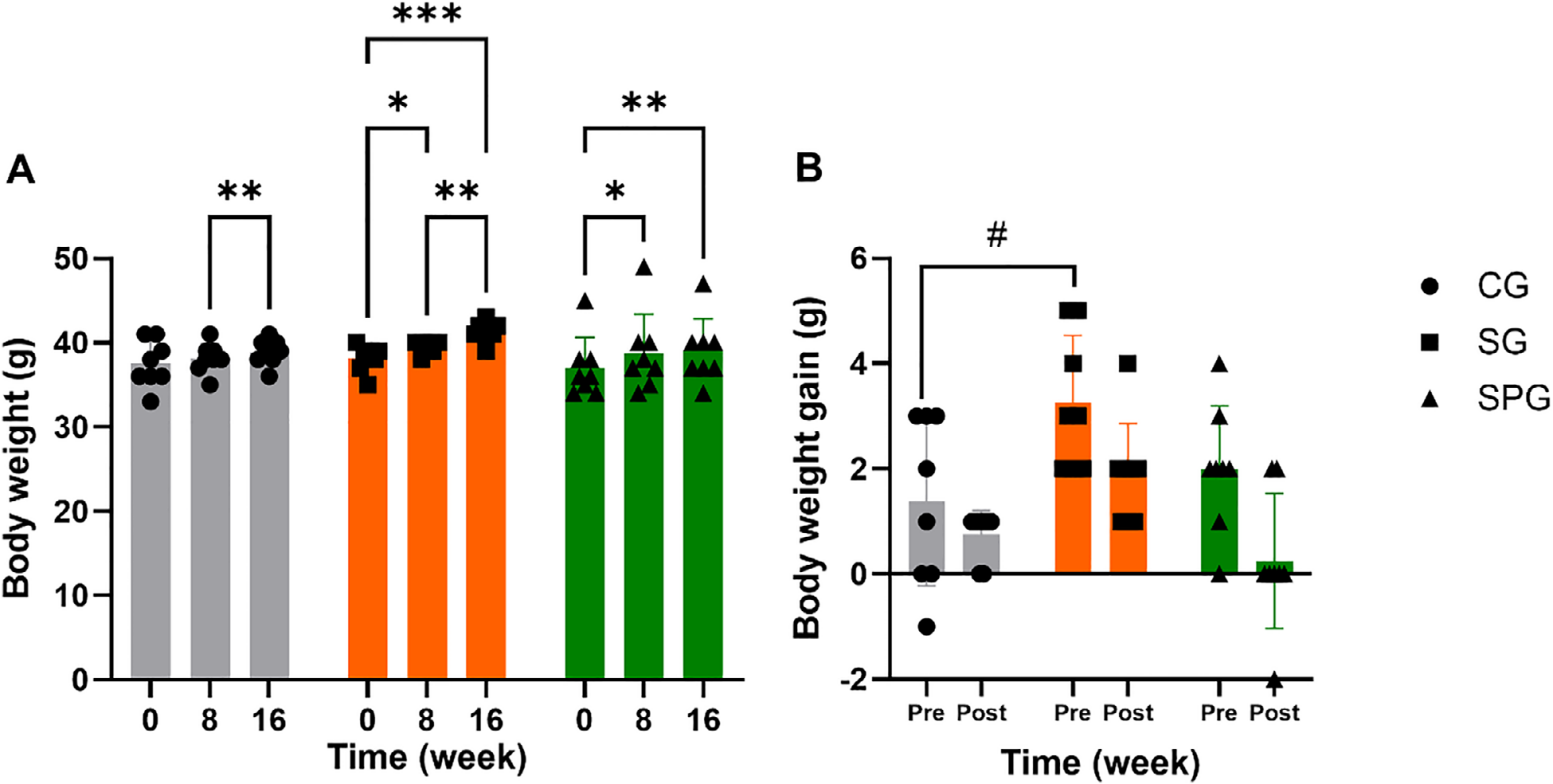
Changes in body weight in the experimental groups. (A) Differences in body weight within groups during the experimental period, (B) differences in body weight between groups and body weight gains. Each experimental group consisted of eight animals (*n* = 8), and data are presented as mean ± SD. Longitudinal body weight measurements were analyzed using two‐way repeated‐measures ANOVA followed by Bonferroni‐adjusted post hoc multiple‐comparison tests. Differences in body weight gain between groups were analyzed using a two‐way ANOVA followed by Bonferroni‐corrected post hoc tests for multiple comparisons. Pre: Preintervention, baseline to week 8. Post: Postintervention, week 8 to week 16. *p < 0.05, **p < 0.01, ***p < 0.001. ^#^ *p* < 0.05. CG: Control group, SG: Shifted group, SPG: Shifted and probiotic supplement group.

Body weight measurements collected longitudinally throughout the experiment were analyzed by time (week) and experimental group (Figure 2). In CG, the difference in body weight between the eighth and 16th weeks was found to be statistically significant (*p* = 0.0062). In the SG, the differences between baseline and eighth week (*p* = 0.0192), baseline and 16th week (*p* = 0.0005), and 8th and 16th weeks (*p* = 0.0026) were found to be significant. Additionally, weight changes between baseline and 8th week (*p* = 0.0389) and week 16 (*p* = 0.0052) in the SPG group were also significant (Figure 2A).

To distinguish the effects before and after probiotic supplementation, body weight progression was additionally evaluated according to preintervention (baseline–week 8) and postintervention (week 8–week 16) phases. Prior to probiotic supplementation, both SG and SPG demonstrated increased body weight gain compared with CG during the preintervention phase. Following initiation of probiotic supplementation at week 8, the increase in body weight observed in the SPG group appeared attenuated compared with the SG group during the postintervention phase. Consistent with this observation, significant differences in body weight gain were detected between CG and SG at the pre‐intervention (*p* = 0.0081) and between SG and SPG at the post‐intervention (*p* = 0.0052) (Figure 2B). In addition, individual animal values were incorporated into the graphical presentations to improve visualization of biological variability and distribution patterns within each experimental group.

**FIGURE 3:**
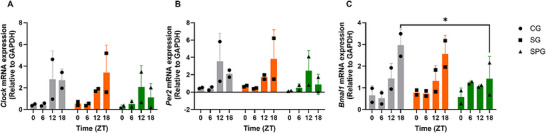
Effects of probiotic supplementation on circadian rhythm disruption in brain tissue caused by altered light/dark cycles. (A) *Clock*, (B) *Per2*, and (C) *Bmal1* gene expression levels in brain tissue were determined by quantitative qRT‐PCR. A total of eight animals were included in each experimental group. Brain tissues were collected from two independent animals per group at each ZT point (ZT0, ZT6, ZT12, and ZT18) (biological replicates, *n* = 2 per group per time point). Each biological sample was analyzed in technical triplicate, resulting in a total of 72 qRT‐PCR reactions analyzed (2 × 4 × 3 × 3). Technical replicates were averaged prior to statistical analyses, and only biological replicates were treated as independent observations. Data are presented as mean ± SD. Gene expression differences among groups and ZT points were analyzed using two‐way ANOVA followed by Bonferroni‐adjusted post hoc testing for multiple comparisons. Because of the limited number of biological replicates per ZT point, the findings should be considered exploratory. *p < 0.05. CG: Control group, SG: Shifted group, SPG: Shifted and probiotic supplement group.

Because gene expression analyses were performed using two independent biological replicates per group at each ZT point, and different animals were used for each ZT sampling point, the statistical outcomes should be interpreted as exploratory findings with limited statistical power. The effects of probiotic supplementation and circadian disruption caused by the altered light/dark cycle on the expression levels of circadian rhythm genes *Clock, Per2*, and *Bmal1* were determined (Figure 3). No statistically significant differences were found in *Clock* expression levels between CG, SG, and SPG at four different time points (ZT0, ZT6, ZT12, and ZT18) (*p* > 0.05). *Clock* gene expression peaked at ZT12 in CG and SPG, while it peaked at ZT18 in SG. A statistically significant difference was found between CG and SPG in terms of *Bmal1* expression levels at ZT18 (*p* = 0.045, Figure 3C).

Scatter‐based graphical presentations including individual biological replicate values and mean ± SD overlays were used to facilitate assessment of within‐group variability and distribution of gene expression data across ZT points.

## 4 Discussion

To our knowledge, evidence regarding the effects of probiotic supplementation on circadian clock gene expression under light/dark shift conditions remains limited, and this study provides preliminary data in this relatively underexplored area.

This study evaluated the effects of circadian rhythm disruption and probiotic supplementation on body weight changes and circadian rhythm gene expression. Although overall body weights were similar between groups throughout the experiment, significant differences in weight gain were observed at some time points, indicating that circadian rhythm disruption may cause weight gain, and that probiotic supplementation may have a partial and limited effect on this outcome. Although there were no significant differences between the groups in circadian rhythm gene expression, differences in peak expression time suggest that circadian disruption may be associated with phase shifts in gene expression patterns. Furthermore, a significant difference in Bmal1 gene expression between the CG and SPG groups at time point ZT18 indicated that probiotic supplementation may have a time‐specific and limited effect on circadian gene response.

In our study, the standard 12‐h light‐12‐h dark cycle was reversed every 2 weeks for 4 months and the shift work model was implemented by taking the example from the study of Khalyfa et al. (2017) [19]. This model was chosen to represent chronic and repeated circadian misalignment under environmental conditions. In addition to exploring the potential use of probiotics as a therapeutic option for circadian disruption, this study provides data on changes in the expression of core clock genes under altered light/dark cycles. However, this study does not provide direct mechanistic or therapeutic evidence, but rather observational molecular data under an environmental circadian disruption model. This study applied an environmentally based circadian disruption model (shift work) to examine misalignment resulting from an environmental factor rather than mutation of specific clock genes. This is because it can simulate the situation in our contemporary society, where circadian disruption is primarily attributed to the environment rather than genetic disruption of the circadian clock gene.

A number of genetic and environmental studies linking the circadian clock and metabolic gene networks are ongoing. In mice, mutations in the circadian clock gene Clock can lead to obesity and alterations in the circadian expression of metabolic genes [20], while in humans, shift work has been associated with increased body mass index [21]. However, probiotic supplementation has been shown to be associated with a partial reduction in weight gain [22, 23]. *Bifidobacterium breve* CCFM1025 strain has been reported to improve body weight and weight loss in sleep‐deprived mice [24]. This is attributed to the fact that metabolites derived from *Bifidobacterium breve* CCFM1025 can influence circadian‐related signaling pathways, including melatonin‐related mechanisms [24]. The findings of this study are partially consistent with previous literature but should be interpreted cautiously due to the limited and nonsignificant nature of several gene expression outcomes. In our study, circadian rhythm disruption was found to be effective in weight gain in the SG and SPG groups compared to the control group (*p* <0.01). Specifically, although mice in the SG group showed the greatest weight gain, mice in the SPG group showed less body weight gain compared to the SG group. This suggests that probiotics may be associated with modulation of weight gain in mice with disrupted circadian rhythms.

A healthy and balanced gut microbiota also plays a critical role in maintaining the host's circadian rhythms [10, 25]. Reciprocal deletion of host circadian clock genes has also been shown to lead to dysbiosis in the gut microbiota [26, 27, 28]. In the absence of microbiota (germ‐free state), the expression patterns of both central and peripheral clock genes (especially the liver clock) are disrupted. The rhythmic expression of core clock genes such as Bmal1 and Per, and clock output regulators such as Dbp, Tef, and Bhlhb42, is altered, resulting in shifts in rhythmic periods. These findings suggest that the gut microbiota is a key modulator in the rhythmic regulation of clock genes and clock output effectors [29]. The present results of gene expression changes in case of circadian rhythm disruption in our study are consistent with those reported in previous studies on mice [15, 30]. Regarding probiotic supplementation, a study conducted on mice with circadian rhythm disruption due to sleep deprivation determined that *Bifidobacterium longum* CCFM1238 exhibited a rhythm‐regulating effect similar to melatonin in vitro and attenuated both intestinal and hypothalamic clock gene disruption (Bmal1, Clock, Per3, Cry1, and Rev‐erbα) [31]. Additionally, in a sleep deprived mouse study, it was determined that sleep deprivation led to irregular circadian gene expression (Bmal1, Clock), and *Lactobacillus kefiranofaciens* K6 supplementation regulated this situation [32]. Our data revealed that altering the light/dark cycle resulted in differences, although not statistically significant, in the expression levels of *Clock*, *Bmal1*, and *Per2* genes in the SG and SPG at four different time periods compared to the CG. Therefore, the nonsignificant differences in our study suggest that probiotic supplementation does not appear to strongly modulate core clock gene expression, but may contribute to subtle or time‐dependent variations. However, a shift in the peak times was observed in the Clock gene (CG/SPG: ZT12, SG: ZT18), and a significant difference was found in Bmal1 expression between CG and SPG at ZT18. This suggests that the probiotic supplementation may exert a limited, time‐specific influence on circadian gene responsiveness.

Although the circadian mechanism in mammals is evolutionarily well‐conserved and therefore very informative when it comes to understanding the fundamental molecular mechanisms, it is difficult to compare nocturnal animals with humans, who evolved as diurnal animals and have a radically different sleep‐wake cycle. Furthermore, animals used in circadian studies are kept under strict 12:12 light‐dark cycles, and experiments are conducted in a controlled environment. Humans have unique lifestyles that further increase the complexity. It is important to consider these factors in human studies.

## 5 Conclusion

Taken together with the current results, these findings suggest that synchronization between circadian and metabolic processes plays an important role in regulating energy balance and body weight control. Overall, altered light/dark cycles have been shown to impact both body weight dynamics and circadian rhythm gene expression patterns. It was concluded that probiotic supplementation may partially modulate both weight gain and changes in certain genes. These observed effects of probiotic supplementation are considered promising for guiding future study designs and for regulating circadian rhythms and achieving positive health outcomes. Therefore, these results provide valuable insights into the potential use of probiotics in the future treatment of circadian rhythm disorders in humans.

However, an important limitation of the present study is the small number of biological replicates used for gene expression analyses at each ZT point (*n* = 2 mice per group). Although technical triplicates were performed to improve measurement reliability, the limited number of independent biological replicates per ZT point (*n* = 2) substantially reduced the statistical power of the circadian gene expression analyses. Therefore, the molecular findings should be interpreted cautiously and considered preliminary. Future studies using larger sample sizes and expanded temporal sampling strategies are needed to confirm the effects of circadian disruption and probiotic supplementation on clock gene expression patterns.

In addition, the study was conducted exclusively in male mice, which may limit the generalizability of the findings given known sex differences in circadian and metabolic regulation; therefore, future studies including both sexes are warranted.

Accordingly, while the present findings suggest that probiotic supplementation may influence circadian rhythm–related molecular pathways and body weight regulation, these observations should be regarded as exploratory rather than definitive evidence for therapeutic application in humans.

## CRediT authorship contribution statement

**Tepebaşı, B**: investigation, animal experiment, methodology, data curation; **Ünlü Söğüt, M**: conceptualization, investigation, animal experiment, supervision; **Kabalı, S**: formal analysis, data analysis, writing – original draft. **Çelik, M.N**: animal experiment, methodology, writing – original draft, writing – review & editing.

## Funding

This study was supported by Ondokuz Mayıs University with the project number PYO.SBF.1904.21.010.

## Conflicts of Interest

The authors declare no conflict of interest.

## Data Availability

The data that support the findings of this study are available on request from the corresponding author. The data are not publicly available due to privacy or ethical restrictions.
